# Temporal Patterns in Hospital Performance Indicators in the Chilean Public Hospital System: A Longitudinal Analysis Using Dimensionless Coefficients

**DOI:** 10.3390/healthcare14142102

**Published:** 2026-07-14

**Authors:** José Rodríguez, Manuel Vargas, Pilar Cos, Andrés Viveros

**Affiliations:** 1 Facultad de Ciencias Económicas y Administrativas, Universidad Católica de la Santísima Concepción, Concepción 4090541, Chile; jorodriguez@ucsc.cl; 2 Escola de Doctorat, Universitat de Lleida, 25003 Lleida, Spain; pilar.cos@udl.cat; 3 Industrial Engineering Department, University of Santiago de Chile, Avenida Victor Jara 3769, Santiago 9170124, Chile; manuel.vargasg@usach.cl

**Keywords:** hospital performance indicators, dimensionless coefficients, temporal stability, Chilean healthcare system, machine learning, healthcare management

## Abstract

**Objectives**: To examine temporal patterns in hospital performance indicators within the Chilean public system between 2019 and 2023 using dimensionless coefficients, which allow comparisons between hospitals of different sizes and case-mix. **Methods**: Data from 3,803,275 discharges across 68 Chilean public hospitals were evaluated in this study. Three dimensionless indicators (relative efficiency, severity, and mortality) were constructed using DRG FONASA fields. The analysis approach is based on descriptive time trends, within-hospital coefficient of variation (CV), IQR-based outlier identification, and RF predictive modeling with time-series cross-validation. **Results**: Most hospitals (95.6%) showed low within-hospital variability (CV < 0.5). The mean efficiency proxy increased from 0.3051 (95% CI: 0.3044–0.3057) in 2019 to 0.3134 (95% CI: 0.3127–0.3142) in 2023. Lagged REP was the strongest predictor in the model (85.1% variable importance). The Spearman correlation between the proposed REP and a VRS input-oriented DEA model (using total length of stay as input and total DRG weight as output) was 0.7857 (*p* < 0.001), indicating a strong convergent association. However, the DEA model employed is limited to a single input and a single output, as data constraints precluded more complex specifications. **Conclusions**: The proposed dimensionless indicators showed relatively stable temporal patterns across most hospitals. The framework may support descriptive longitudinal comparisons across heterogeneous institutions, though external validation is required.

## 1. Introduction

Assessing hospital performance is an important challenge for public health systems, particularly amid budget constraints and rising demand for care. The system of public hospitals in Chile has around 68 hospitals for more than 15.5 million FONASA users (average 2020–2023; FONASA open data [1]) and discharges approximately 760,000 people per year. It operates on fixed budgets and is subject to geographic and case-mix variations among its providers. In Chile, the Diagnostic-Related Groups (DRG) system has been used as a basis for resource allocation, with limited incorporation of broader institutional performance dimensions beyond service volume.

DRGs group patients into categories based on clinical characteristics and the resources they are expected to consume, and are used extensively for budgeting and cost containment, as well as for comparative management applications [2]. The National Health Fund (FONASA) in Chile gradually adopted DRG-based linked payment mechanisms, with full implementation in January 2020. The study period 2019–2023 encompasses the early development stage of the DRG system and the major operational disruption associated with the COVID-19 pandemic. Therefore, this dataset, which reflects the country, gives us a chance to study patterns of hospital performance across systemic changes. Recent international evidence shows that DRG-based systems are increasingly used not only for reimbursement, but also as tools for hospital performance monitoring, cost control, and comparative management. Liu and Ye [3], using DRG data from 130 tertiary hospitals in Sichuan, showed that DRG-derived indicators such as case-mix index, number of DRG groups, total weight, time efficiency, mortality in low-risk groups, and standardized fatality rates can be integrated into multidimensional hospital performance evaluation models. Similarly, Wang et al. [4] proposed a comprehensive performance evaluation system under DRG payment reform that combines indicators for fund management, medical service capability, medical cost control, reimbursement, and satisfaction. These studies support the relevance of DRG-based administrative data for comparative performance assessment, while also highlighting the need for composite and context-sensitive approaches.

### 1.1. Limitations of DRG-Based Evaluations

While DRGs provide a standardized framework for classifying patients by expected resource use, they have well-documented limitations for comprehensive performance assessment. First, DRGs capture clinical complexity primarily through administrative codes, which may be subject to upcoding or variation in coding practices across hospitals. Second, DRGs do not directly measure quality of care, patient outcomes (beyond mortality), or patient satisfaction. Third, DRG-based evaluations may not fully account for social determinants of health, socioeconomic conditions, or geographic accessibility—factors that substantially influence hospital performance in publicly funded systems like Chile’s. Fourth, DRG systems typically focus on inpatient care, omitting outpatient and preventive services that are integral to population health. For these reasons, the proposed framework does not claim to replace multidimensional performance assessment but rather to complement existing approaches by offering a stable, scale-normalized indicator for longitudinal descriptive monitoring. At the same time, recent evaluations of DRG payment reforms indicate that improvements in cost control or length of stay should not be interpreted uncritically as improvements in quality or technical efficiency. Li et al. [5], in an interrupted time-series analysis of DRG reform in Meishan, China, found reductions in hospital costs, patient cost-sharing, and length of stay, but no statistically significant effect on 30-day readmission rates, suggesting that efficiency-related indicators require parallel quality monitoring. Likewise, Li et al. [6] reported that DRG reform reduced hospitalization costs across hierarchical hospitals, although its effects varied by hospital grade and produced limited structural optimization. These findings reinforce the need to interpret DRG-based performance indicators as descriptive proxies rather than definitive measures of quality, efficiency, or managerial effectiveness.

Data Envelopment Analysis (DEA), a methodology extensively documented in the international literature, has been widely used to evaluate hospital performance [7]. When comparing heterogeneous public hospitals over time, DEA may be sensitive to model specification, variable selection, and contextual assumptions, despite its usefulness [8,9]. When the goal is to assess longitudinal performance trajectories rather than competitive positioning, complementary approaches may also be helpful in publicly funded systems like Chile’s. These methodological and contextual considerations support the exploration of complementary indicators for longitudinal comparison in public hospitals. Accordingly, this study applies a framework based on dimensionless coefficients to the Chilean public hospital system during 2019–2023. The study addressed three questions: whether hospital rankings were consistent over time, whether the proposed indicators were temporally stable within hospitals, and whether prior values of the efficiency proxy were associated with subsequent values in exploratory predictive models.

### 1.2. Bridging the Gap: Why a Complementary Framework Is Needed

Given the variable selection problem, the difficulty of comparing units at different scales, and the presence of unobserved inputs and outputs (which are well-known issues in DEA that parallel other nonparametric analyses), it is doubtful whether DEA can be used for longitudinal comparison across heterogeneous hospitals. The proposed framework overcomes these limitations in three concrete ways. First, using ratio-based normalization, the dimensionless coefficients become less dependent on the absolute scale, enabling a hospital of a different size to be compared without imposing a priori variable-selection decisions. Second, in contrast to competitive ranking, the approach emphasizes individual temporal trajectories within hospitals and is descriptive, stability rather than frontier estimation driven. Third, by explicitly distinguishing the REP as a proxy for technical efficiency (rather than a measure of it), the framework refrains from making causal assertions about inefficiency. Hence, the outlined technique should be considered a descriptive tool for longitudinal monitoring, not a replacement for DEA when frontier estimation is feasible.

### 1.3. Practical Contributions of the Proposed Framework

Compared with established hospital efficiency methodologies, the proposed framework offers several practical advantages. First, it requires only three DRG-derived fields (weight, severity, mortality) and length of stay, making it feasible in settings where detailed input-output data are unavailable. Second, because the indicators are dimensionless, they can be compared across hospitals with different scales and case-mix profiles without extensive variable selection or model specification. Third, the framework prioritizes longitudinal descriptive monitoring over point-in-time ranking, which may be more relevant for internal quality improvement than for competitive benchmarking. For healthcare managers, the indicators could support the identification of hospitals with atypical temporal trajectories, the generation of hypotheses about operational patterns, and low-cost longitudinal tracking. For policymakers, the framework may complement existing performance assessment systems by providing a stable, transparent, and easily communicated set of descriptive indicators. However, the framework should not be used for high-stakes resource allocation or punitive performance evaluation without external validation. Therefore, this study has a single primary objective: to examine temporal patterns in hospital performance indicators within the Chilean public system between 2019 and 2023 using a framework based on dimensionless coefficients. Specifically, we address three research questions: (1) Are hospital rankings consistent over time? (2) Do the proposed indicators show temporal stability within hospitals? (3) Are prior values of the efficiency proxy associated with subsequent values in exploratory predictive models?

### 1.4. Theoretical Framework: Distinguishing Relative Efficiency from Classical Efficiency

In traditional health economics, efficiency is usually defined as the amount of output obtained per unit of input and is estimated using frontier-based techniques, such as Data Envelopment Analysis (DEA) or Stochastic Frontier Analysis (SFA). Such methods view any departure from an ideal frontier as inefficiency. Nevertheless, they are sensitive to the choice of model specification and variables, as well as to the assumption that all relevant inputs and outputs have been observed.

On the other hand, the definition of relative efficiency used here is deliberately more limited. Instead of purporting to be a measure of absolute/technical efficiency, the Relative Efficiency Proxy (REP) is the ratio of the DRG weight (expected complexity) to the length of stay. This measure has no presumption of optimality and does not require a definition of the production frontier. Instead, it is a descriptive, scale-normalized quantity that enables one to compare temporal trajectories across arbitrary hospitals without making any strong parametric assumptions.

The theoretical justification for this approach draws on three strands of literature. First, panel data structures have been shown to control for unobserved heterogeneity and capture temporal dynamics in hospital performance [10]. Second, ratio-based normalization has been successfully applied in comparative efficiency research to reduce dependence on absolute measurement units [9]. Third, dimensionless coefficients are widely used in engineering and systems biology to compare systems of varying scales under shared proportional relationships [11]. Translating this logic to healthcare, the proposed framework is positioned as a complementary descriptive tool rather than a substitute for frontier-based methods.

## 2. Materials and Methods

Previous studies have identified methodological problems in measuring hospital performance. Hollingsworth [12] suggested that although DEA is popular in many fields, its results are sensitive to specification choices and may not generalize to other contexts. One significant limitation noted in the literature is the exclusion of relevant variables related to patient quality and complexity. Rosko and Proenca [10] demonstrate that the omission can have a substantial impact on estimates of hospital efficiency. They explicitly state that “Subsequent estimates of hospital inefficiency may be enhanced by employing panel data, which would permit a more precise estimation of hospital X inefficiency as well as by incorporating superior measures of quality and outcomes. Patient outcomes, severity of the disease, and more detailed information about the outpatients should be included” (p. 77). Specialized literature also independently acknowledges the potential benefits of panel data structures for deriving more robust comparative estimates, as they control for unobserved heterogeneity and incorporate time dynamics [10]. Meanwhile, several voices have emerged advocating the creation of standardized measures that, if they came to exist, could enable more uniform comparisons of heterogeneous nonprofit organizations.

Authors such as Kohl et al. [13], in their extensive review of the literature on HCMS, explicitly emphasize the need for methodological innovation to improve the comparability and practical applicability of efficiency studies, noting that “future research should focus on developing metrics that are less sensitive to model specifications and more useful to managers” (p. 245). Within the literature reviewed for this study, limited evidence was identified regarding frameworks that combine longitudinal analysis with simple, dimensionless indicators explicitly intended for cross-hospital comparison.

Accordingly, this study applies a longitudinal analytical framework that combines panel-based temporal structure with dimensionless coefficients to improve interpretability across hospitals with heterogeneous scales and case mixes. The purpose is to examine whether this formulation is useful for descriptive comparison and temporal monitoring within the available dataset.

This was a quantitative, retrospective, longitudinal study based on anonymized records from the FONASA DRG system for the period 2019–2023. Dimensionless Coefficients: Conceptual Rationale. Heterogeneity in size, case mix, and service structure complicates comparative assessment of hospital performance. Absolute metrics such as total cases, beds, or expenditures are difficult to compare directly across institutions. Data on hospital expenditures or detailed resource consumption were not available in the administrative dataset. Therefore, this study uses ratios without physical units to standardize selected relationships between DRG-based complexity, length of stay, severity, and mortality. This approach reduces scale dependence in descriptive comparisons. Hospital administrative data were normalized using a ratio-based method in the current study. The Diagnosis-Related Groups (DRG v3.4) system-based central technical fields from the FONASA dataset are used to accomplish this. A code like 29301 in this system is made up of a Major Diagnostic Category (such as “29” for circulatory diseases), a DRG type, a particular group code, and a final digit that indicates the case’s severity level (from 0 for “no severity” to 3 for “highest severity”). A Relative Weight (IR_29301_WEIGHT), which indicates the anticipated clinical complexity and related resource consumption, is assigned to each combination.

Operational Definitions (with technical fields from the FONASA dataset):

RELATIVE_EFFICIENCY_PROXY (REP) = IR_29301_WEIGHT/STAY

Interpretation: This ratio was used as a descriptive proxy intended to relate case complexity to hospitalization time. Higher values may reflect shorter stays for comparatively complex cases; however, they may also be influenced by discharge practices, coding patterns, patient selection, referral structure, bed availability, and other unmeasured organizational factors. Therefore, REP should not be interpreted as a direct measure of technical efficiency or care quality.

RELATIVE_SEVERITY = IR_29301_SEVERITY/IR_29301_WEIGHT

Interpretation: This ratio, used here as a descriptive indicator of case-mix composition, combines the recorded severity level with the DRG weight. Because the relationship between coded severity and DRG weight is partly embedded in the classification system and may not always be an independent indicator of clinical burden, values should be interpreted with caution.

RELATIVE_MORTALITY = IR_29301_MORTALITY/IR_29301_WEIGHT

Interpretation: This ratio was intended to summarize mortality in relation to DRG-based case complexity. Its interpretation depends on the exact construction of the underlying mortality field in the FONASA database. Therefore, in this manuscript, it should be treated as a comparative outcome-related indicator rather than as a validated risk-adjusted quality measure unless the observed-versus-expected specification is explicitly documented.

The methodological justification is also in line with recent literature indicating that DRG systems should be treated with caution when addressing within- and between-group heterogeneity. Cai et al. [14] present a tree-structured multi-objective optimization framework for forming DRGs that accounts for intragroup homogeneity and intergroup heterogeneity, as well as interpretability and grouping constraints. While the present analysis is not an attempt to reconstruct DRG groups, this body of literature supports the more general thesis that DRG-based indicators must account for clinical complexity, the heterogeneity inherent in resource use within DRGs, and the shortcomings of straightforward, unadjusted comparisons. The method to be introduced is ratio normalization, which is well-suited for descriptive comparisons among hospitals operating at different scales and with different case-mix distributions. First, the coefficients are dimensionless indicators that may reduce direct dependence on the original measurement units, a concern raised in the comparative efficiency literature [9]. Second, each coefficient is multiplied by a variable, IR_29301_PESO, which captures expected case complexity, either in the numerator or the denominator.

Ratio-based normalization methods are employed throughout the sciences to describe associations without dependence on absolute magnitude. Dimensionless coefficients are widely employed in engineering to relate systems of different sizes that are subject to the same proportional relationships [11]. Here, we aim to see whether similar normalization logic improves descriptive comparability across hospitals with different organizational setups. The proposed coefficients were designed as a pragmatic comparative tool for analyzing institutional variance using administrative data. These are additional descriptive indicators, not definitive measures of hospital efficiency or replacements for techniques such as DEA [15,16].

### 2.1. Data Cleaning Procedure

The original FONASA database contained 4,709,438 discharge records for the period 2019–2023. Records with missing or invalid hospital identifiers, missing DRG codes, non-positive length of stay (STAY ≤ 0), non-positive DRG weight (IR_29301_WEIGHT ≤ 0), or inconsistent severity-mortality coding were excluded. After applying these filters, the final analytical dataset included 3,803,275 valid records (80.8% of the original), representing 68 hospitals and 95 medical specialties.

**Figure 1:**
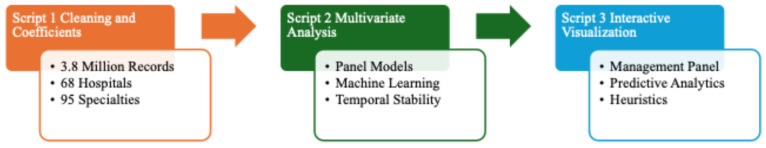
Analysis Pipeline. Source: Prepared internally based on FONASA data (2019–2023).

The reproducible pipeline implemented in Python 3.10.11 is shown in Figure 1, which summarizes the analytical workflow from data cleaning to indicator construction, statistical analysis, predictive modeling, and dashboard visualization. This pipeline was designed to improve methodological transparency by making each stage of the analysis traceable and reproducible. The specific role of each script is described in the following subsection.

After data cleaning, the analytical dataset included 3,803,275 valid records from 68 hospitals and 95 specialties. Preliminary descriptive analyses suggested relatively low within-hospital variation in the proposed efficiency proxy indicator over the study period. Detailed quantitative findings are presented in the Section 3.

### 2.2. Reproducibility and Analytical Workflow

To improve reproducibility, the complete analytical workflow was organized as a sequential Python-based pipeline. The anonymized dataset and the three Python scripts used to reproduce the analyses are available in the public repository indicated in the Data Availability Statement. The repository includes the analytical dataset and scripts.

The workflow was implemented in three scripts. Script 1 performs data verification, data cleaning, construction of the dimensionless indicators, and robustness analyses. Starting from the original FONASA DRG discharge-level database for 2019–2023, which contained 4,709,438 records, the script verifies the availability of the main analytical fields, including hospital identifier, medical specialty, year, length of stay, DRG weight, DRG severity, and DRG mortality-related variables. Records with missing or invalid hospital identifiers, missing DRG codes, non-positive length of stay, non-positive DRG weight, or inconsistent severity-mortality coding are excluded. After applying these criteria, the final analytical dataset comprises 3,803,275 valid records, representing 80.8% of the original database, across 68 hospitals and 95 medical specialties. The script then computes the three dimensionless indicators used in the study: the Relative Efficiency Proxy, calculated as DRG relative weight divided by length of stay; the Relative Severity indicator, calculated as DRG severity divided by DRG relative weight; and the Relative Mortality indicator, calculated as the DRG mortality-related field divided by DRG relative weight. Script 1 also aggregates the cleaned discharge-level data into hospital-year observations and estimates descriptive yearly trends, within-hospital coefficients of variation, interannual ranking persistence, and year-by-year IQR-based outliers. Script 2 generates the interactive dashboard visualization. Using the cleaned dataset and the computed indicators, the script prepares hospital-specialty-year observations for 68 hospitals, 95 medical specialties, and the full 2019–2023 period. For visualization purposes, the dashboard prioritizes 20 reference hospitals selected according to care volume and clinical diversity. The resulting HTML panel includes interactive hospital-level visualizations, a hospital-selection menu, and longitudinal displays of the proposed indicators. This script does not introduce additional analytical filters beyond those applied in the cleaning stage; its purpose is to translate the analytical outputs into an exploratory visualization interface for descriptive monitoring.

Script 3 performs predictive and exploratory machine learning analyses. The script prepares a hospital-year predictive dataset of 260 observations from 65 hospitals covering 2020–2023, using lagged indicator values and hospital characteristics derived from the cleaned data. A Random Forest model is trained and evaluated on 2023 data. The script reports prediction error metrics, feature-importance values, classification metrics based on a study-specific high-performance threshold, and exploratory operational heuristics. In the corrected run, lagged REP was the most important predictor, and the correlation between current REP and lagged REP was 0.938, supporting the interpretation of temporal persistence within the analyzed sample. These predictive outputs are interpreted as exploratory and hypothesis-generating rather than as causal evidence or externally validated forecasting tools.

Together, the three scripts reproduce the full analytical sequence of the study: data verification, data cleaning, indicator construction, hospital-year aggregation, temporal stability analysis, IQR outlier detection, dashboard visualization, predictive modeling, classification analysis, DEA comparison, PCA, sensitivity analyses, and generation of interactive outputs. This structure was designed to allow independent replication of the analyses using the dataset and scripts provided in the public repository.

### 2.3. From Data to Decision: An Interactive Panel for Healthcare Management

As a supplementary output of the analytical workflow, an interactive dashboard prototype was developed to display hospital-level indicators across years, specialties, and selected performance dimensions. In this manuscript, the dashboard is presented as a visualization interface derived from the study outputs, not as a validated decision-support tool. No formal usability testing or reliability assessment has been conducted. Therefore, the dashboard should be interpreted as an academic prototype for illustrative purposes only. To focus the descriptive display on higher-volume institutions, the panel prioritizes 20 reference hospitals that meet dual criteria of care volume (≥1000 cases) and clinical diversity (≥3 specialties) (Table 1).

### 2.4. A Concrete Bridge Between Academia and Management: The Interactive Panel Prototype

**Figure 2:**
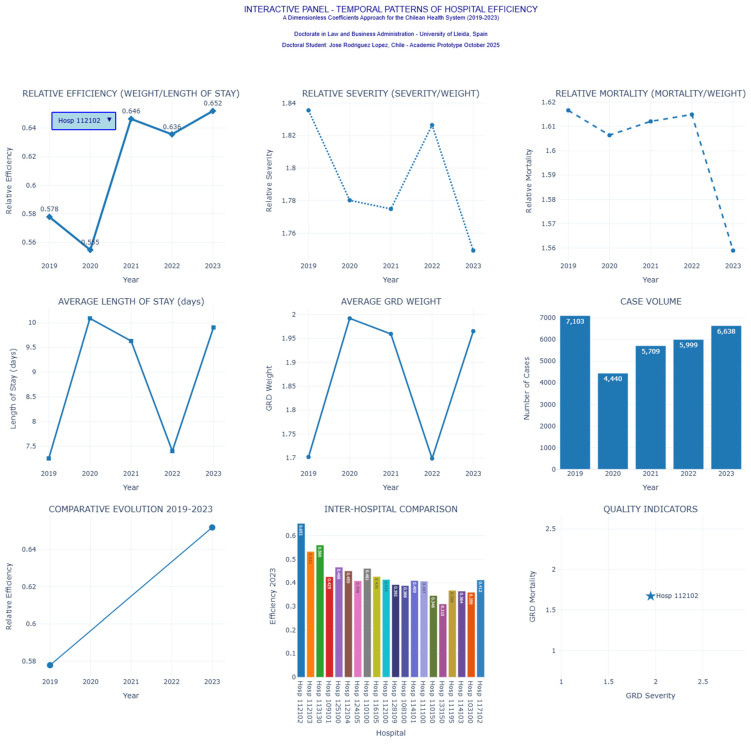
View of the interactive management panel prototype. Note: The tool consolidates nine key metrics of efficiency, severity, and outcomes for 68 Chilean hospitals (2019–2023).

To translate the proposed framework into a practical management tool, an interactive dashboard prototype was developed to support longitudinal monitoring of the proposed hospital indicators. The prototype enables quasi-real-time tracking of proxy indicators, descriptive identification of deviations, and decision-making based on contextualized historical evidence rather than isolated cross-sectional observations. In this sense, the tool is intended to transform accountability into a continuous and formative process, allowing managers to detect emerging deviations before they become entrenched. Figure 2 illustrates the dashboard prototype, which consolidates nine key metrics related to efficiency, severity, and outcomes for 68 Chilean public hospitals during 2019–2023.

### 2.5. Mathematical Foundations of the Models

#### 2.5.1. Temporal Stability Analysis: Foundations and Limitations

##### Conceptual and Methodological Foundations

An essential component of assessing the consistency of hospital performance is temporal stability analysis. Our longitudinal approach using the intra-hospital coefficient of variation (CV) enables us to characterize the operational volatility of each facility over the five-year period 2019–2023, in contrast to cross-sectional approaches that capture efficiency at a particular point in time. The coefficient of variation (CV) was selected as the primary metric for describing within-hospital variability because it normalizes dispersion relative to the mean and provides a scale-independent measure that can be compared across hospitals with different sizes and complexity profiles. For each hospital h, the CV was calculated as:

$$CV_{h}=\frac{\sigma_{h}}{\mu_{h}}$$

where $\sigma_{h}$ represents the standard deviation of the proposed proxy indicator for hospital h during 2019–2023, and $\mu_{h}$ represents the corresponding mean value of the same indicator over that period. The index h denotes the unique hospital identifier, ranging from 1 to 68.

### 2.6. Epidemiological and Operational Context

From an epidemiological and operational perspective, the dataset reflects a heterogeneous hospital population and a broad mix of clinical activity. The original database included 4,709,438 discharge records from 2019 to 2023. Women accounted for 59.0% of records and men for 41.0%, with a mean age of 44.3 years (SD 25.5) and a median age of 43.8 years. Emergency admissions represented the most frequent admission type, and the leading ICD-10 codes were I10, Z37.0, and E11.9. The most represented specialties were Obstetrics and Gynecology, General Surgery, and Internal Medicine, with a mean length of stay of 6.0 days. This clinical and demographic heterogeneity supports the need for cautious interpretation of hospital comparisons based only on administrative indicators.

This analysis is particularly relevant given the historical context under review. The period 2019–2023 encompasses a pre-pandemic phase of normal operations (2019), two years of maximum disruption due to COVID-19 (2020–2021), and a period of recovery and a new normal (2022–2023). In this scenario, the within-hospital CV can serve as a descriptive indicator of temporal variability over a period that included a major system disruption. Lower variability may reflect greater consistency.

### 2.7. Interpretation and Classification Criteria

For descriptive expressions, CV values were categorized according to empirically determined criteria. Values  <  0.1 were interpreted as very low variability, indicating little variation in the suggested proxy-indicator over time. A value of 0.1  <  v  <  0.5 was interpreted as moderate variability, while values  ≥  0.5 were considered as high variability. We regarded values > 1.0 as very high variability and analyzed them for possible atypical temporal patterns. These divisions were employed solely to simplify descriptive interpretation and should not be taken as prescriptive or indicative thresholds for good or bad hospital performance.

### 2.8. Added Value for Healthcare Management

Finding that 95.6% of hospitals had CV values below 0.5 suggests little variation in the proposed efficiency proxy indicator across hospitals over time. Such a pattern could indeed be consistent with long-term surveillance and the detection of hospitals whose trajectories deviate strongly from the entire sample, but it does not constitute proof of the intervention’s effectiveness, the reliability of forecasting, or the quality of management. From an organizational perspective, this temporal stability could serve to provide guidance related to budget planning based on past trajectories, inform the development of interventions in terms of attainable and sustainable goals and in terms of expectations of sustainability (sequence of stages including anomalies), as well as to identify exceptional establishments warranting special consideration. This procedure marks a departure from snapshot efficiency evaluation to a dynamic description of indicator trajectories, with the understanding that temporal stability may matter for descriptive monitoring and longitudinal planning.

### 2.9. Detection of Outliers Using IQR: Statistical Robustness and Contextual Application

#### Methodological Basis and Technical Justification

Detection of outliers also plays a key role in the descriptive analysis of hospital performance indicators, as it allows the separation of random variation from potentially complex, highly atypical observations. The interquartile range (IQR) method was chosen in the present work because it is robust to outliers and less influenced by asymmetric distributions, which is of utmost importance given the non-parametric nature of the suggested proxy indicators. The IQR rule is used to establish outlier boundaries based on the data’s percentile distribution. The interquartile range equals:

IQR = Q3 − Q1

where Q1 is the 25th percentile and Q3 the 75th percentile of the indicator distribution. Then the lower and upper bounds are given by:

Lower limit = Q1 − 1.5 × IQR(1)

Upper bound = Q3 + 1.5 × IQR

The scaling factor 1.5 is traditionally used to detect moderate outliers and guard against their influence on the threshold definition.

### 2.10. Choice of Univariate IQR over Multilevel Models

Even though multilevel/hierarchical models might more adequately account for variability across institutions and for nested data structures (patients nested within hospitals, which are nested within years), the current study uses a univariate IQR for two pragmatic reasons. First, the main purpose is to describe and identify outliers to generate hypotheses, not to perform causal inferences that would require adjustment for nested random effects. Second, the small number of hospital-year observations (260) limits the complexity of multilevel models that can be stably estimated.

Nevertheless, the limitations of univariate approaches are acknowledged: IQR does not account for hospital-level random variation or temporal autocorrelation. Future research should extend this descriptive analysis by using multilevel models with appropriate random-effects specifications. The current univariate approach should be interpreted as an initial exploratory screen rather than a definitive classification of outliers.

### 2.11. Methodological Advantages in the Healthcare Context

The IQR approach was chosen over other methods, such as Z-score-based methods, for methodological and practical reasons. First, unlike methods based on standard deviation, the IQR is computed from percentiles and is less affected by outliers when estimating dispersion [17]. This characteristic is especially valuable in hospital data, as a few hospitals may have extreme values due to case-mix composition, coding practices, referral patterns, or organizational factors. Second, the IQR is applicable to skewed distributions, which is important given that hospital proxy efficiency indicators are likely to be skewed and may not meet parametric assumptions [18]. Third, quartile-based thresholds are simpler and easier to explain to healthcare managers than model-based statistical cutoffs. Lastly, the approach enables descriptive notice of both abnormally high and abnormally low values of an indicator, each of which could serve as a cue for further contextual examination rather than as a definitive label of superior or inferior performance.

### 2.12. Application and Validation Protocol

The outlier protocol was conducted annually from 2019 to 2023. To account for temporal changes in the distribution of the suggested indicators, particularly during the pandemic period, the thresholds were determined individually for each year. To avoid interpreting temporary fluctuations as meaningful deviations, a hospital’s outlier status was defined as persistent if the hospital was identified as an outlier in more than one period, considering three or more years as a persistent outlier. Moreover, outlying results were compared with hospital case volume, and results driven by insufficient annual sample sizes were not considered robust signals. For hospitals with persistent atypical results, further exploratory analyses are performed: medical specialty, predominant case complexity, and contextual factors are examined to explain the findings.

### 2.13. Limitations and Critical Considerations

When applying IQR-based outlier detection in this context, a few caveats should be kept in mind. In low-volume hospitals, slight variations in case mix can lead to seemingly aberrant values; for this reason, a minimum case-volume threshold was implemented to reduce the likelihood of false-positive classifications. Furthermore, the IQR is a univariate technique, which means it analyzes each indicator on its own and thus cannot identify intricate multivariate patterns. For this reason, additional descriptive and multivariate analyses were performed to complement the previous analyses. Also to be considered is the arbitrary nature of the 1.5 × IQR threshold. However, this threshold may not be appropriate across all medical fields, so it is recommended to conduct sensitivity analyses using different thresholds to assess the robustness of the findings. Lastly, IQR-based detection also does not inherently differentiate among transient disruption, sustained improvement, structural rigidity, or enduring inefficiency. Thus, outlier status should be viewed as signaling that further scrutiny is warranted rather than as an endpoint classification of hospital performance.

### 2.14. Potential Management Use

From a supervisory perspective, detecting outliers using the IQR may serve as a descriptive, reinforcing element in longitudinal surveillance. It is possible that organizations with exceptionally high scores are outliers worthy of further organizational examination, particularly to see if their trajectories reflect better (or worse) practices, case-mix influences, coding, or contextual factors. Conversely, below-average hospitals may also be subject to scrutiny to determine whether they face unrecognized operational bottlenecks, data quality problems, or institutional dysfunctions. At the system level, the prevalence and persistence of outliers may indicate the extent of among-hospital heterogeneity and warrant attention to hospitals for further qualitative or operational assessment. In this respect, the detection of outliers should be interpreted as a means of generating hypotheses that support monitoring and the formulation of inquiries, and not as an end for benchmarking hospitals, sanctioning, or ranking them.

### 2.15. Random Forest Predictive Model: Algorithmic Fundamentals and Application in Hospital Proxy Efficiency

Random Forest was then applied as an exploratory predictive model to test whether non-linear combinations of lagged proxy efficiency and other associated variables could better predict future efficiency values [19]. Because relatively few hospital-year observations are available to train such a model, the machine-learning results should be viewed as predictive exploration rather than conclusive evidence of mechanisms. A Random Forest model was used.

$${\hat{y}}_{i}=\frac{1}{B}\sum_{b=1}^{100}T_{b}(x_{i})$$

In this expression, ŷᵢ denotes the predicted proxy-indicator value for hospital i, while B represents the total number of trees in the ensemble. In the final model, B was determined through cross-validation. The term Tᵦ(xᵢ) denotes the prediction produced by the b-th decision tree for the vector of hospital characteristics xᵢ, which includes historical values of the proxy efficiency indicator, severity, and mortality-related indicators.

### 2.16. Model Architecture and Hyperparameters

The final Random Forest parameterization was selected through cross-validation, balancing predictive performance and model parsimony. A grid search was conducted over the following hyperparameters: number of trees (n_estimators = 50, 100, and 200), maximum tree depth (max_depth = 5, 10, and 15), and minimum number of samples required to split an internal node (min_samples_split = 2, 5, and 10). The optimal configuration selected by cross-validation was n_estimators = 50, max_depth = 5, and min_samples_split = 2. Bootstrap sampling was used so that each decision tree was trained on a random sample of the observations, thereby increasing diversity across trees and reducing model instability. Because the predictive dataset included only 260 hospital-year observations, the model was interpreted as exploratory rather than as an externally validated forecasting tool. Furthermore, at each split, a random selection of features is drawn, and the best split is found among them, considering the $\sqrt{p}\;$p predictors for classification problems. This procedure decorrelates the trees and yields more stable exploratory predictions.

### 2.17. Validation of Statistical Assumptions

Robustness checks were conducted to characterize the statistical features of the data under investigation and to inform the interpretation of subsequent models. The Augmented Dickey–Fuller test could not reject the null hypothesis of a unit root (ADF = −0.939, *p* = 0.775), indicating potential non-stationarity in the REP series. The Breusch-Pagan test detected heteroscedasticity (LM = 55.288, *p* < 0.001), as expected due to differences in variability among hospitals. The Shapiro–Wilk test indicated deviations from normality (W = 0.989, *p* = 0.018), so non-parametric methods were also applied. Finally, the constructed indicators exhibited a high degree of multicollinearity, as indicated by the maximum variance inflation factor (max VIF = 450.08), which is perhaps unsurprising given that multiple measures are derived from DRGs. These results together suggest that parametric models should be treated with caution and indicate the utility of flexible, exploratory methods such as Random Forest.

### 2.18. Model Validation and Diagnostics

To assess potential overfitting given the limited sample (260 hospital-year observations), time-series cross-validation was performed using Timeseries Split with 3 folds, respecting the temporal order of the data. Grid search was conducted over the following hyperparameters: n_estimators (50, 100, 200), max_depth (5, 10, 15), and min_samples_split (2, 5, 10).

The optimal configuration was n_estimators = 50, max_depth = 5, min_samples_split = 2, with an average RMSE of 0.0016 across folds. Feature importance was stable across folds, with lagged REP (EFFICIENCY_LAG1) consistently ranked first (mean importance: 85.1%, standard deviation: 0.0011). Despite these precautions, the model remains exploratory. Prospective validation on new data would be required before any operational forecasting use.

## 3. Results

### 3.1. Temporal Stability Analysis: Descriptive Evidence of Relative Stability

The temporal stability analysis, based on the methodological framework of dimensionless coefficients, was applied to a consolidated database of 3,803,275 valid records (80.8% of the original total), covering the operation of 68 hospitals and 95 medical specialties in the Chilean public system during the period 2019–2023. This dataset provides broad longitudinal coverage of public hospital activity during the study period [13].

**Figure 3:**
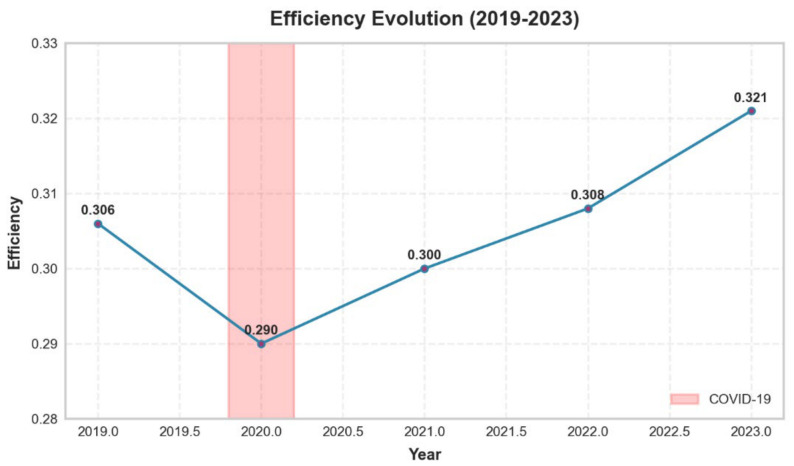
Temporal Evolution of Efficiency Proxy Indicator.

As shown in Figure 3, the mean efficiency proxy indicator increased slightly over the study period after the decline observed during the early pandemic phase. This pattern is consistent with temporal recovery in the proposed indicator, although the observational design does not allow attribution of the change to specific organizational adaptations or system-level resilience.

### 3.2. Intra-Hospital Coefficient of Variation: Descriptive Variability Analysis

**Figure 4:**
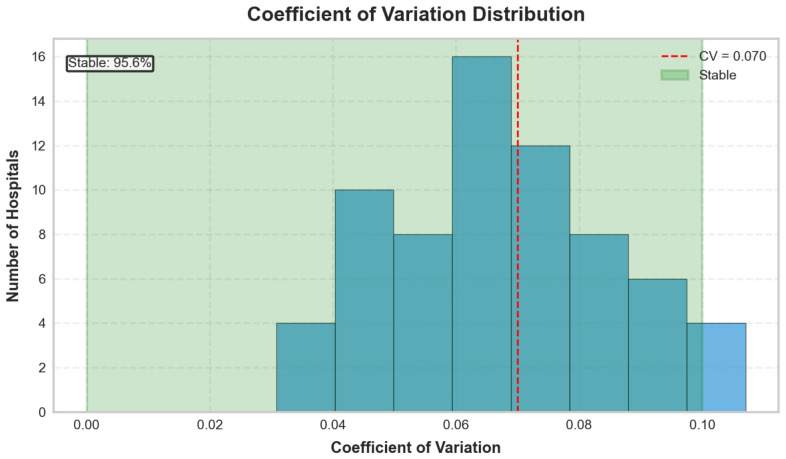
Distribution of Intra-Hospital Coefficients of Variation.

The CV within each hospital was calculated to quantify the variability of the proposed proxy indicator within hospitals over the study period. Overall, the results suggested modest dispersion across hospitals. More specifically, 65/68 (95.6%) hospitals had a CV < 0.5, and 63/68 (92.6%) had a CV < 0.1. The mean CV for the entire sample was 0.059. These findings, which are also collected in Figure 4, reveal that the proposed proxy indicator exhibited relatively minimal change within most hospitals over time, though this trend is to be viewed as descriptive and should not be taken as evidence of on-the-ground stability or of managerial competence.

The low observed dispersion indicates that the proposed proxy indicator changed relatively little within most hospitals across the study period, including the pandemic years. This may reflect persistent operational patterns and relatively low variability across hospitals during the analyzed period [20]. This pattern is compatible with persistent institutional trajectories, but the present analyses do not identify the mechanisms responsible for that consistency. The identified stability is particularly relevant given that it operates in a highly heterogeneous institutional setting, where hospitals of varying complexity, size, and geographic contexts coexist.

This finding may reflect persistent administrative and clinical-operational patterns within the Chilean public system, rather than evidence of specific management mechanisms. The low observed volatility (average CV = 0.059) may be relevant for longitudinal planning, as it suggests that historical trajectories could help establish realistic expectations for future monitoring and intervention assessment.

### 3.3. Sensitivity Analysis: Pre-Pandemic vs. Pandemic Periods

To examine whether the COVID-19 pandemic affected REP values, separate analyses were conducted for three periods: pre-pandemic (2019), pandemic (2020–2021), and post-pandemic (2022–2023). The mean REP values were compared across periods using Mann–Whitney U tests. The mean REP decreased from 0.3051 in 2019 to 0.2934 during the pandemic (2020–2021), representing a relative decline of 3.85% (*p* < 0.001). In the post-pandemic period (2022–2023), the mean REP recovered to 0.3087, a 5.22% increase from the pandemic period (*p* < 0.001). The post-pandemic REP was not statistically different from the pre-pandemic level (*p* = 0.1123). This suggests that the proposed proxy indicator returned to values comparable to the pre-pandemic period, although this should not be interpreted as evidence that overall hospital performance or care quality returned to baseline. These findings indicate that REP values decreased during the pandemic and later returned to levels statistically comparable to those of the pre-pandemic period. However, this pattern should not be interpreted as evidence of full system recovery or resilience, since the indicator does not incorporate clinical quality, readmissions, staffing, or patient-level outcomes.

### 3.4. Caveat: Low Variability May Reflect Rigidity or Inertia

An important caveat is warranted. While low temporal variability may be consistent with operational regularity, alternative interpretations are equally plausible. Low variability may also reflect structural rigidity, where hospitals are unable to adapt their processes despite changes in demand or patient mix. It may indicate bureaucratic inertia, in which entrenched routines persist regardless of opportunities for performance improvement. It may also represent persistent inefficiency, where hospitals remain stuck at suboptimal performance levels without improvement or deterioration. Furthermore, low variability could be an artifact of mathematical compression inherent in ratio-based indicators, particularly if the denominator (DRG weight) and numerator (length of stay) are correlated. Therefore, low CV values should not be interpreted uncritically as evidence of effective management. Instead, they should be viewed as descriptive patterns requiring additional contextual and qualitative investigation.

### 3.5. Predictive Models and Performance Heuristics: Exploratory Performance Patterns

**Figure 5:**
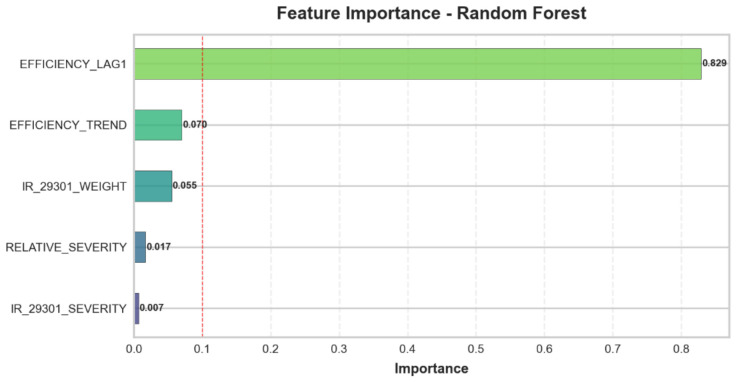
Importance of variables.

A random forest-based predictive analysis was performed on 260 hospital-year data points from 65 hospitals across 2020 to 2023. Lagged REP was ranked the most important predictor in that model, based on the selected variable-importance metric, and current and lagged REP were highly correlated. These results show temporal persistence in the data under the model, but they should not be taken as evidence of causal dominance or of general forecasting performance. This is consistent with the phenomenon of “efficiency inertia”, which is well-documented in the path-dependence literature on healthcare organizations, where prior trajectories strongly anchor subsequent performance [13]. These results are summarized in Figure 5.

When the model was used to predict REP value in 2023 from prior-period data, the reported performance metrics were RMSE = 0.0136 and mean absolute error = 0.0053. These values suggest a close fit within the analyzed sample, but their interpretation is limited by the lack of external validation and the modest number of hospital-year observations. This finding corroborates previous research that identifies “operational momentum” as a potentially relevant correlate of observed performance patterns [20]. The prediction results are shown in Figure 4.

The reported model fit (R^2^ = 0.89) indicates that the selected predictors captured a substantial proportion of variance in the analyzed sample. However, the model should be interpreted as a preliminary forecasting exercise. Its suitability for prospective management use would require external validation and assessment under future operational conditions. The current results support a tentative interpretation of temporal persistence rather than a definitive claim about causal determinants of performance.

**Figure 6:**
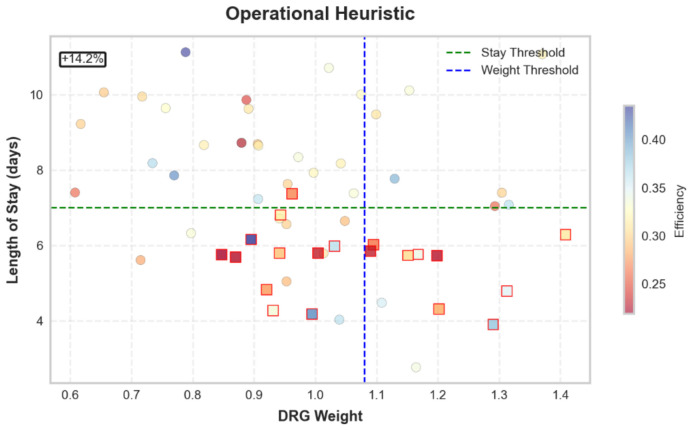
Operational heuristics. Circles represent hospitals in the general sample. Squares represent hospitals that meet the heuristic thresholds (DRG weight ≥ 1.08 and length of stay ≤ 7.0 days). Dashed lines show the thresholds for length of stay (green) and DRG weight (blue).

The dispersion plot in Figure 6 shows that higher observed REP values were concentrated in hospitals that combined relatively higher DRG weights with shorter stays.

The visual pattern is consistent with the descriptive group comparison, but it should be interpreted as exploratory because the graph does not control for confounding factors or specialty composition.

The identified threshold of DRG Weight > 1.08 corresponds to medium-to-high complexity cases, typically elective surgical procedures and medical conditions with significant comorbidities, where process optimization may be particularly relevant. Additionally, a limit of < 7.0 days of stay suggests a potentially favorable operational range in which bed-day use may be comparatively lower for higher-complexity cases, although implications for quality of care cannot be assessed with the available data.

This pattern may be useful as a hypothesis-generating operational profile for further investigation. It suggests that some hospitals with higher-complexity case mixes also achieved shorter stays and higher values of the proposed proxy indicator. Whether this profile is transferable or desirable across settings requires separate clinical and managerial evaluation. The identified 14.2% gap represents a descriptive performance gap within the analyzed sample within the system, particularly relevant in contexts of budget constraints, where optimizing the performance of existing installed capacity is a relevant management consideration [9].

### 3.6. Classification Analysis Using a Study-Specific High-Performance Threshold

Using a study-specific threshold defined at the 75th percentile (>0.315) of the REP distribution in the sample, the classification model achieved an overall accuracy of 89.2%. This threshold is internal to the dataset and has not been externally validated or benchmarked against clinical quality indicators. Therefore, it should not be interpreted as a validated measure of “high performance” or clinical excellence. Instead, it serves as a descriptive cutoff for exploratory classification within this study. Future research should establish clinically meaningful thresholds using external quality standards.

**Figure 7:**
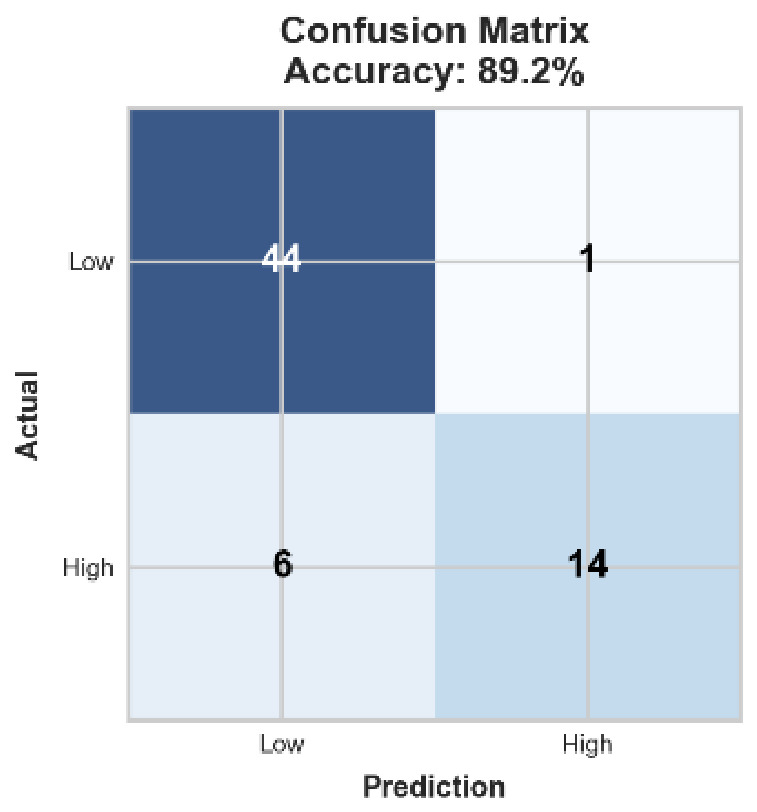
Confusion matrix.

The confusion matrix in Figure 7 indicates that the model had 14 true positives and 0 false positives in the tested sample, with 6 hospitals above the chosen threshold being missed by the model. This trend implies a conservative classification profile, but its value is application-dependent on the relative costs of a false positive and a false negative. Because the threshold is study-specific, the classification output should be interpreted as exploratory rather than as a validated identification of institutional excellence.

### 3.7. Temporal Patterns of Performance Outliers: High-Value Outlier Patterns During the Study Period

**Figure 8:**
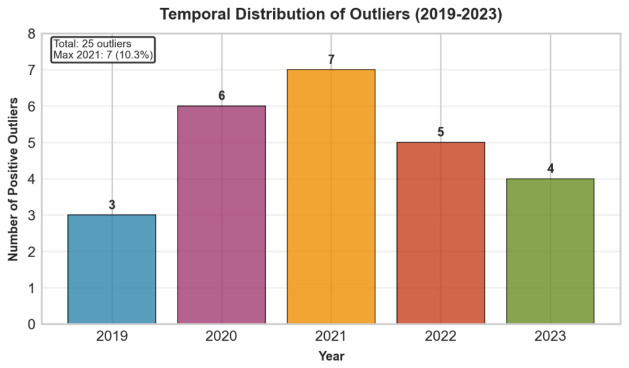
Temporal Patterns of Performance Outliers.

25 hospital-year observations with values above the annual upper outlier threshold between 2019 and 2023 were found by the IQR-based analysis. Rather than direct proof of better institutional performance, these observations should be viewed as candidates for further research, as they show statistically significant values for the chosen indicator. These observations are shown in Figure 8.

The temporal distribution of high outlier values varied across years, with more such observations detected during 2020–2021 than in some adjacent years. Although this pattern shows variation in hospital trajectories during the pandemic, it does not prove that performance was enhanced by the crisis. According to Kruk et al. [21], their identification offers epidemiologically significant case studies for reproducing best practices, which are especially pertinent for handling upcoming medical emergencies.

The temporal distribution of high outlier values suggests heterogeneity in hospital trajectories during the pandemic period. The three hospitals that remained outliers for four consecutive years may be considered recurrent high-value cases that warrant further organizational investigation. However, these observations should not be interpreted as direct evidence of resilience, superior management models, or crisis-driven improvement without additional clinical, operational, and contextual analysis.

### 3.8. Validation of Model Assumptions

The diagnostic tests indicated departures from several assumptions commonly required by simple parametric models, including stationarity, homoscedasticity, normality, and low collinearity. These findings support cautious interpretation of conventional linear-model outputs in this dataset, but they should not be generalized as definitive structural properties of the Chilean hospital system.

The Augmented Dickey–Fuller test failed to reject the null hypothesis of a unit root (ADF = −0.939, *p* = 0.775), suggesting possible non-stationarity in the REP time series. Therefore, the ADF result should not be interpreted as evidence of structured temporal persistence. Evidence of temporal persistence in this study is instead supported by year-to-year correlations and lagged predictive patterns. This pattern is consistent with prior literature suggesting that hospital-level indicators may exhibit temporal inertia due to organizational routines and path dependence [13].

### 3.9. Addressing Multicollinearity: Principal Component Analysis

To assess whether the three dimensionless coefficients measure distinct performance dimensions, a principal component analysis (PCA) was performed on the hospital-year panel data (*n* = 328). The first principal component (PC1) explained 82.21% of the total variance, while PC2 and PC3 explained 15.40% and 2.39%, respectively. The cumulative variance explained by PC1 and PC2 reached 97.61%.

The loadings (component weights) for PC1 were 0.5204 (relative efficiency), −0.5950 (relative severity), and −0.6124 (relative mortality), indicating that all three indicators contribute to a common underlying dimension. However, the remaining 17.79% of variance explained by PC2 and PC3 suggests that each indicator captures some unique information.

These results indicate that while the three indicators are mathematically interdependent due to shared DRG components, they are not fully redundant. For descriptive purposes, reporting all three remains informative. However, for inferential models, using them as simultaneous predictors is problematic due to multicollinearity, as acknowledged in the manuscript.

The noted heteroscedasticity (Breusch-Pagan Test: LM = 55.288, *p* < 0.001) is consistent with institutional heterogeneity, in which the variance of efficiency across hospitals differs significantly. This variation in practice is consistent with what has been described as the radically different nature of public hospital systems, in which contextual and structural elements produce differentiated outcomes [9,20]. The non-normality of residuals (Shapiro–Wilk test: W = 0.989, *p* = 0.018) is consistent with the choice of flexible nonparametric methods like Random Forest, whose procedures are not based on the normality assumption to draw sound inferences. This characteristic might facilitate the use of machine learning methods as complementary tools alongside classic parametric models in complex healthcare scenarios [19,22]. Very high Variance Inflation Factor (VIF) values (max = 450.08) were also observed for the dimensionless coefficients, indicating severe multicollinearity. This outcome was expected because many indices are ratio-based metrics derived from common DRG-related components. Hence, collinearity is more likely to arise from structural mathematical dependence between constructed variables rather than from anomalies in the data. For that matter, these variables should not be considered independent predictors in a traditional linear model, but they may still be useful in descriptive and exploratory analyses [2,23]. Taken together, all these results align well with the analytical assumption we pursued in this study: that a complex system demands analytic tools that grasp, rather than impose, its structural character. These deviations do not invalidate the descriptive analyses, but they support cautious interpretation and reinforce the need for complementary non-parametric approaches in this heterogeneous hospital network with interrelated performance dimensions.

Stochastic Frontier Analysis (SFA) was also attempted, but did not converge to a solution with meaningful variability. While this non-convergence may be partially attributable to the low temporal variability in the data, alternative explanations are equally plausible: model misspecification, multicollinearity among candidate predictors, or insufficient variation across hospitals to reliably estimate the stochastic parameters. Therefore, the non-convergence of SFA should not be interpreted primarily as evidence of structural system stability. Instead, it underscores the challenges of applying parametric frontier methods to these data and supports the use of non-parametric and descriptive approaches in this context (Table 2).

### 3.10. Sensitivity Analysis: Alternative DEA Specifications

To assess whether the convergent validity between the REP and DEA was sensitive to model specification, two additional DEA models were estimated using the available data. Model 2 included total length of stay and number of discharges as inputs, with total DRG weight as the output. Model 3 used the total length of stay as input, with total DRG weight and number of discharges as outputs. The Spearman correlations between the REP and these alternative DEA specifications were 0.5816 for Model 2 and 0.8134 for Model 3, both statistically significant (*p* < 0.001). The primary specification, Model 1, yielded a Spearman correlation of 0.7857. These results indicate that the association between REP and DEA estimates was sensitive to model specification, ranging from moderate (ρ = 0.58) to strong (ρ = 0.81). Nevertheless, all correlations were statistically significant, suggesting that REP captures a related, although not identical, performance dimension across different DEA specifications.

## 4. Discussion

### 4.1. Implications for Chilean Healthcare Management

The results suggest persistent temporal patterns in the proposed indicators across the analyzed hospitals. The main contribution of this study is the application of a standardized ratio-based framework to a large administrative dataset, allowing assessment of whether these indicators generate interpretable longitudinal patterns in this setting. Formal validation against external standards remains pending.

It is important to highlight that the REP is not proposed as a direct measure of technical or allocative efficiency, but rather as a descriptive proxy for comparing temporal patterns across hospitals. Shorter lengths of stay do not necessarily indicate better quality or higher efficiency, as they may be influenced by discharge practices, patient selection, or coding patterns.

A comparative analysis with DEA provided additional empirical support for the proposed framework. Despite a moderate rank correlation (Spearman ρ = 0.7857), the proposed dimensionless indicator showed substantially lower within-hospital variability (CV = 0.0592 vs. 0.0944) and higher global persistence (Spearman ρ = 0.860 vs. 0.744). The Pearson persistence of the proposed indicator reached 0.938, indicating strong temporal inertia. These differences are expected because DEA is more sensitive to annual fluctuations and outliers, whereas the ratio-based indicator, by normalizing DRG weight, may filter some random variation. The comparison does not aim to replace DEA but rather to position the dimensionless coefficients as a complementary, stable descriptive tool for longitudinal monitoring. The inability of SFA to produce convergent estimates highlights the difficulty of applying parametric frontier models to this dataset, particularly given the limited number of hospital-year observations, high multicollinearity among constructed indicators, and restricted availability of conventional input variables. This result should not be interpreted as evidence of structural stability in the Chilean hospital system, but rather as a methodological limitation that supports cautious interpretation and the use of complementary descriptive and non-parametric approaches.

It should be noted that the DEA model employed is limited to a single input (total length of stay) and a single output (total DRG weight), as data constraints precluded more complex specifications. Future research with access to hospital-level data on staffing, infrastructure, and operating costs would be needed to assess whether the convergent validity observed here holds under more comprehensive DEA specifications.

Traditional methods such as Data Envelopment Analysis (DEA) remain useful and widely applied, although prior studies have noted potential sensitivity to specification choices, variable selection, and contextual assumptions in some comparative settings [8,20]. Other authors have highlighted the potential value of panel structures for controlling unobserved heterogeneity and examining temporal dynamics [10,13].

The present study combines longitudinal analysis with dimensionless coefficients intended to reduce direct scale dependence in hospital comparisons. Rather than replacing established methods, the framework should be interpreted as a complementary descriptive approach that may be useful when the objective is to examine temporal regularities in administrative data from heterogeneous institutions. The predictive models provide additional support for temporal persistence in the analyzed data, using the Random Forest algorithm, recognized for its ability to capture complex and nonlinear relationships without restrictive parametric assumptions [19]. Lagged REP contributed strongly to model predictions, which is consistent with the idea that past REP values are associated with subsequent REP values in this sample. This finding should be interpreted as evidence of persistence, not as proof of a universal organizational law.

Based on these exploratory findings, the clearest pattern observed in the predictive analysis is temporal persistence within the analyzed sample. Historical efficiency accounted for 82.9% of the selected variable-importance metric, suggesting that prior values were strongly associated with subsequent values in the model. This result should be interpreted as evidence of within-sample persistence rather than as proof of a universal organizational mechanism.

### 4.2. Distinguishing Statistical Persistence from Meaningful Healthcare Performance

It is important to clarify that the temporal persistence observed in the proposed indicators (e.g., correlation of 0.938 between REP values in consecutive years) reflects statistical association and inertia in the measured proxy. This persistence does not necessarily indicate improved healthcare quality, enhanced patient outcomes, or operational effectiveness. Hospitals with consistently low REP values may be persistently inefficient rather than stable in a positive sense. Conversely, hospitals with stable high REP values may simply maintain favorable case-mix and discharge practices without delivering better clinical care. Therefore, statistical persistence should not be equated with effective management or quality improvement. The findings are descriptive and hypothesis-generating, not evaluative.

Several limitations should be considered. First, the study relies on administrative data and therefore cannot directly measure relevant organizational factors such as staffing composition, leadership, referral networks, discharge support, or institutional culture. Second, the 2019–2023 period includes major disruptions associated with the COVID-19 pandemic, which may affect coding practices, case mix, and length of stay in ways not fully captured by the proposed indicators. Third, the predictive models were developed from a relatively small number of hospital-year observations and were not externally validated. These issues limit causal interpretation, generalizability, and operational deployment.

From a governance perspective, DRG-based indicators should also be understood as part of broader public management reforms. Simonet [24], analyzing the French experience, argues that DRGs have functioned not only as accounting tools, but also as instruments of performance monitoring, fiscal discipline, standardization, and central steering. This perspective is relevant for interpreting the proposed framework as a descriptive monitoring tool rather than as a stand-alone mechanism for evaluating institutional quality or policy success.

### 4.3. Broader Implications and Future Research Agenda

This study presents a case study of the Chilean public hospital system, but the conceptual model could be applied to other middle-income countries with similar DRG-based administrative data systems. Dimensionless coefficients could also be employed to examine descriptive trends in longitudinal monitoring, in countries undergoing health system reforms, or in developing performance-based purchasing systems, where frontier methodologies are not applicable due to data or model-specification constraints. Further research could externally validate the framework using DRG data from other countries, such as Colombia, Peru, or European health systems, to assess its generalizability across nations. Future work should address criterion validity by evaluating REP trajectories against independent quality measures, such as risk-standardized mortality rates, patient-reported experience measures, readmission rates, or accreditation scores. Furthermore, the strength of and changes in policy, including DRG payment reform, in relation to REP trajectories could be analyzed using quasi-experimental methods, e.g., instrumental variables or difference-in-differences, to test whether policy shifts predict changes in REP trajectories. Finally, prospective implementation studies may determine whether dashboard-based monitoring is operationally useful by introducing the tool to hospital management teams and assessing its impact on their decision-making and longitudinal monitoring.

### 4.4. Omitted Variable Bias and Unmeasured Confounders

The relevance of important clinical and organizational characteristics should lead to a discussion on whether omitted variable bias played a role. Information on nurse-to-patient ratios, readmission rates, patient satisfaction scores, and the quality of outpatient follow-up was not available in the administrative dataset. If these unmeasured factors are correlated with both DRG weight and length of stay, they might confound the observed associations. For instance, a “hospital” with shorter stays and higher readmission rates might appear more “efficient” under the REP when the shorter stays are driven by post-discharge complications. On the other hand, an institution with long stays and good outpatient follow-up might be disadvantaged by the measure. Although temporal stability analyses suggest persistent hospital-specific patterns, we cannot rule out confounding by unmeasured variables. To determine whether the REP reflects operational efficiency or merely captures discharge and coding practices, future work linking DRG data with clinical registries and patient-reported outcomes is required.

### 4.5. Practical Applicability for Policymakers and Hospital Managers

It is important to clarify how the proposed framework could be operationalized in practice, while acknowledging current limitations. The indicators are best suited for low-stakes descriptive monitoring, such as identifying hospitals with atypical temporal trajectories for further investigation, generating hypotheses about operational patterns, or complementing existing dashboards with a longitudinal perspective. They are not suitable for high-stakes decisions such as resource allocation, punitive performance evaluation, or hospital ranking without external validation. Specific barriers to immediate operational use include the lack of external validation, reliance on internally derived thresholds (e.g., the 75th-percentile cutoff), and limited assessment of clinical significance. Therefore, the manuscript recommends that interested policymakers or hospital administrators first conduct pilot implementations with qualitative feedback loops before relying on the indicators for decision-making. Future research should focus on establishing clinically meaningful thresholds, validating them against external quality indicators, and assessing their impact on management decisions through controlled implementation studies.

### 4.6. Methodological Limitations and Considerations

When interpreting the coefficient of variation (CV) analysis, several methodological caveats must be kept in mind. First, the CV may be distorted by extreme values, particularly in short time series, such as the five-year period considered in this study. To overcome the problem, the variability analysis was supplemented with IQR-based outlier detection. Second, the magnitude, but not the direction, of variation can be measured by the CV. Hence, a hospital that is continuously improving and another that is continuously becoming worse could have the same CV. Third, the coefficient of variation is mean-dependent and may be inflated when the mean is close to zero. This risk was minimized by dropping observations with non - positive mean values. Fourth, Overall variability can be represented by the CV, but specific patterns in the time domain, such as seasonal change, nonlinear trajectories, and directional trends, cannot be captured by the CV. For these reasons, the analysis was completed using interannual correlation analysis and time-series models. Finally, the observed stability should be regarded in the context of the pandemic era, as the Chilean public hospital system operated under extraordinary guidelines throughout 2020–2021, which conceivably altered coding practices, case mix, length of stay, and the normal flux of hospital activity.

## 5. Conclusions

This study suggests that ratio-based indicators may offer a useful complementary option for examining temporal patterns in hospital administrative data when direct comparison of absolute metrics is problematic.

The comparative validation with DEA revealed a Spearman correlation of 0.7857, a lower within-hospital CV (0.0592 vs. 0.0944), and a markedly higher global persistence (Pearson r = 0.938 for the proposed indicator vs. Spearman r = 0.744 for DEA), supporting the convergent validity and temporal consistency of the proposed dimensionless framework.

Additional support for the proposed framework comes from the predictive analyses, in which lagged REP was the most influential predictor within the modeled sample. Together with the high correlation between current and prior REP values, this result is consistent with temporal persistence in hospital-level indicator values. However, it should not be interpreted as formal validation of the framework, as causal evidence, or as proof of a general law governing hospital performance.

These findings suggest that the proposed indicator may exhibit relatively persistent trajectories over time. For this reason, future analyses of hospital improvement initiatives should consider longer observation periods rather than relying only on short-term fluctuations in the indicator. However, the design and evaluation of specific interventions require additional clinical, organizational, and policy evidence beyond the REP-based framework.

Overall, this study provides an applied example of combining longitudinal analysis and ratio-based indicators in a public hospital dataset. The framework may be useful for descriptive monitoring and hypothesis generation when direct comparison of absolute metrics is difficult. However, further comparative and external validation is required before broader methodological or policy claims can be sustained.

## 6. Limitations

### Methodological Considerations

Deviations from parametric assumptions identified here should be considered empirical characteristics of the data under analysis rather than individual statistical anomalies. These results warrant caution in relying on simple parametric models, argue for the use of more flexible analytical methods, and moderate the strength of inference obtainable from any single model specification. Heteroscedasticity is justified because different procedures are performed in different hospitals, while non-normality can be addressed with complementary non-parametric techniques such as Random Forest. Additionally, the substantial multicollinearity observed among the constructed indicators reflects the inherent dependence among the suggested efficiency-, severity-, and mortality-related dimensions, as these dimensions derive from DRG-based components. Altogether, these findings emphasize the importance of caution when interpreting results from conventional parametric models in the context of this data set.

## Figures and Tables

**Table 1:** Referral hospitals with significant patient volume. Source: Prepared internally based on FONASA data.

Hospital	Proxy Indicator of Relative Efficiency	Cases	Specialties
112102	0.620	29.327	32
112103	0.543	16.082	11
113130	0.513	31.523	23
109101	0.488	31.156	31
125100	0.449	35.835	52

**Table 2:** Comparison of metrics between the proposed dimensionless efficiency proxy and DEA (VRS input-oriented).

Metric	Proposed Indicator	DEA
Spearman correlation with proposed indicator	1.000	0.7857
Mean within-hospital CV	0.0592	0.0944
Global persistence (Spearman)	0.860	0.744
Global persistence (Pearson)	0.938	—

Note: DEA persistence is reported only using Spearman’s rank correlation because the Pearson correlation assumes linearity, which may not hold for DEA scores. The proposed indicator shows higher stability and persistence.

## Data Availability

The anonymized dataset and Python scripts required to reproduce the analyses are openly available at: [https://drive.google.com/drive/folders/1YcQC8P0EaHwAoGhxPEZo9AUOPc0uZdHA], accessed on 22 June 2026. The repository includes the analytical dataset, three Python scripts for data cleaning and indicator construction, statistical and predictive analyses, and dashboard visualizations.
